# Carboxylate derivatives of tributyltin (IV) complexes as anticancer and antileishmanial agents

**DOI:** 10.1186/s40199-017-0174-0

**Published:** 2017-04-04

**Authors:** Durdana Waseem, Arshad Farooq Butt, Ihsan-ul Haq, Moazzam Hussain Bhatti, Gul Majid Khan

**Affiliations:** 1 Department of Pharmacy, Quaid-i-Azam University, Islamabad, 45320 Pakistan; 2 Department of Chemistry, Allama Iqbal Open University, H-8, Islamabad, 44000 Pakistan

**Keywords:** Organotin (IV), Anticancer, Antileishmanial, ADMET, Protein kinase inhibition

## Abstract

**Background:**

Tributyltin (IV) compounds are promising candidates for drug development. In the current study, we evaluated in-vitro and *in-silico* profile of carboxylate derivatives of tributyltin (IV) complexes.

**Methods:**

ADMET and drug-likeliness properties were predicted using MetaPrint2D React, preADMET, SwissADME and Molsoft tools. SwissTargetPrediction predicted molecular targets for compounds. In-vitro bioactivity was evaluated by quantifying cytotoxicity against HepG2, THP-1 cell lines, isolated lymphocytes and *leishmania* promastigotes as well as measuring protein kinase (PK) inhibition activity.

**Results:**

Results indicate partial compliance of compounds with drug-likeliness rules. Ch-409 complies with WDI and Lipinski rules. ADMET profile prediction shows strong plasma protein binding except for Ch-409, low to high GI absorption and BBB penetration (C_brain_/C_blood_ = 0.942–11; caco-2 cells permeability 20.13–26.75 nm/sec), potential efflux by P-glycoprotein, metabolism by CYP3A4, medium inhibition of hERG, mutagenicity and capacity to be detoxified by glutathionation and glucuronidation. Molecular targets include proteases, enzymes, membrane receptors, transporters and ion channels where Ch-409 targets membrane receptors only. Compounds are significantly (*p* < 0.05) cytotoxic against HepG2 cell line and *leishmania* as compared with normal isolated lymphocytes. Ch-459 indicates highest toxicity against *leishmania* (mortality 97.9 ± 3.99%; LC50 0.323 ± 0.002 μg/mL) whereas Ch-409 possesses maximum cytotoxicity against HepG2 cell line (IC50 0.08 ± 0.001 μg/mL) as well as 97.5 ± 1.98% (LC50 0.954 ± 0.158 μg/mL) mortality of *leishmania* promastigotes. It was observed that antileishmanial effect was reduced by 16.38%–34.38% and 15–38.2% in the presence of NaN_3_ and mannitol respectively. PK inhibition and reactive oxygen species production are possible mechanisms for cytotoxicity.

**Conclusions:**

Selected carboxylate derivatives of tributyltin (IV) complexes possess significant antileishmanial and cytotoxic potential. These are promising compounds for the development of antileishmanial and anticancer drugs.

**Graphical Abstract:**
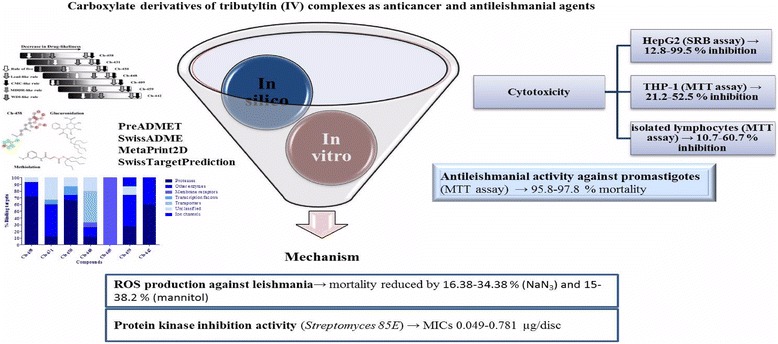
Carboxylate derivatives of tributyltin (IV) complexes as anticancer and antileishmanial agents

## Background

Cancer and leishmaniasis are major threats to humans causing significant morbidity and mortality worldwide. Leishmaniasis is caused by transmission of *leishmania* parasite through sand fly in both endemic and non-endemic areas [1]. Lack of effective measures to control both parasite and sand fly are major factors for the spread of disease. Current therapies are inadequate to manage cancer and leishmaniasis due to diversity of molecular disruptions and development of resistance respectively. Cancer evolves by deregulating endogenous functions of molecular proteins, which in turn can be targeted to impede cancer progression. Anticancer drugs are developed and investigated against angiogenesis, extracellular matrix proteins and a variety of signal transduction pathways, including mitogen-activated protein kinase (MAPK), Janus-activated kinase, transforming growth factor-beta, p53, Ras, Wnt and Akt signaling [2, 3]. Treatment strategies against leishmaniasis involve killing parasite by DNA fragmentation, formatting aqueous pores in promastigotes cell membrane, oxidative mitochondrial damage, decreasing mitochondrial membrane potential, affecting peptidases that constitute *leishmania* genome and disrupting kinases responsible for *leishmania* division and differentiation [4]. Glycogen synthase kinase that control leishmania cell cycle, is a new potential target for antileishmanial drugs [5].

Regression of cancer and leishmaniasis is a challenge that can be accomplished by developing efficacious and cost-effective drugs. Among multiple synthetic drugs, organometallic compounds are prospective candidates for anticancer and antileishmanial drug discovery [6, 7]. Organometallics including pentavalent antimonials [8] and platin derivatives [9] have been used for over three decades for the management of leishmaniasis and cancer respectively [10]. Sodium stibogluconate and meglumine antimoniate are first line drugs against all forms of leishmaniasis [11]. On the other hand, carboplatin, oxaliplatin and cisplatin are commonly employed metal based drugs against ovarian, breast, head/neck, bladder, lung and colorectal cancers [12]. Efficacy of these compounds is compromised due to substantial risk of toxicities and emergence of resistance [8, 12]. Clinical limitations and inadequate control of subject diseases demand to investigate new drugs. It is general consensus that there are other metals in periodic table with therapeutic potential. Structural diversity and redox and catalytic properties of organometallics make them promising drug candidates. Among these, organotin (IV) compounds have caught the attention of researchers for their prospective biocidal activities. Carboxylate derivatives of organotin (IV) compounds have been previously investigated for their anticancer and antileishmanial profile [10]. Novel tin based compounds have been characterized with proven antibacterial, antifungal and antitumor activities [13, 14]. Considering the growing importance of organotin (IV) compounds in medicine, the present study was designed to evaluate the cytotoxic potential of tributyltin (IV) compounds against cancer cells and *leishmania*. The rationale was to appraise metal based drugs that can be effective and efficacious in managing rapidly spreading cancers and leishmaniasis. We assessed *in-silico* drug-likeliness, ADMET profile and in-vitro anticancer and antileishmanial activities of new carboxylate derivatives of tributyltin (IV) ligand.

## Methods

### Chemicals, cell lines and strains

Standards including surfactin, amphotericin B and vincristine were procured from Sigma-Aldrich (Steinheim, Germany). Doxorubicin was purchased from Merck (Darmstadt, Germany). Medium ISP4 for protein kinase (PK) assay was prepared in the laboratory. Unless stated otherwise, all other chemicals were purchased from Sigma-Aldrich (Germany). Human leukemia (THP-1) (ATCC # TIB-202) and human hepatoma (HepG2) (RBRC-RCB1648) cell lines were used for cytotoxicity assays. *Streptomyces* 85E and *Leishmania tropica* kwh 23 were used for protein kinase inhibition and antileishmanial assays respectively.

### Compounds

**Fig. 1:**
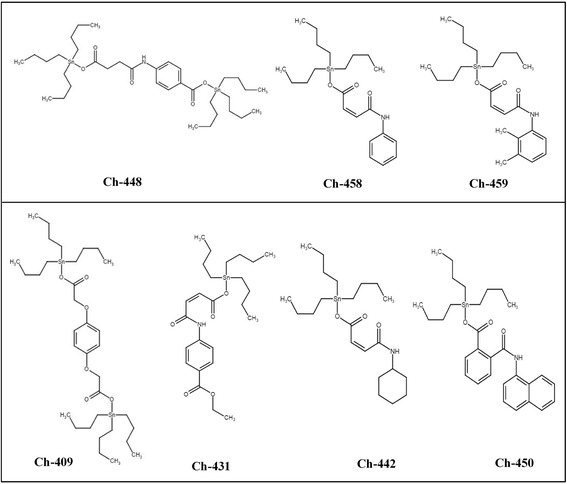
Structures of tributyltin (IV) compounds

Carboxylate derivatives of tributyltin (IV) backbone were selected from library of synthetic compounds present at our laboratory (Fig. 1). These compounds were selected based on structural similarity to compounds previously reported to possess cytotoxic profile [10]. These included bis(tributylstannyl) 2,2’-(1,4-phenylenebis(oxy))diacetate (Ch-409), ethyl (Z)-4-(4-oxo-4-((tributylstannyl)oxy)but-2-enamido)benzoate (Ch-431), tributylstannyl (Z)-4-(cyclohexylamino)-4-oxobut-2-enoate (Ch-442), tributylstannyl 4-(4-oxo-4-((tributylstannyl)oxy)butanamido)benzoate (Ch-448), tributylstannyl 2-(naphthalene-1-ylcarbamoyl)benzoate (Ch-450), tributylstannyl (Z)-4-oxo-4-(phenylamino)but-2-enoate (Ch-458) and tributylstannyl (Z)-4-((2,3-dimethylphenyl)amino)-4-oxobut-2-enoate (Ch-459). Synthesis and characterization data on these compounds are submitted for publication elsewhere.

### *In-silico* screening

#### Drug-likeliness prediction

PreADMET and Molsoft tools were utilized to determine the drug-likeliness of tributyltin (IV) compounds and some marketed drugs [15]. PreADMET calculates drug-likeliness of compounds based on Lipinski rule, lead-like rule, CMC-like rule, MDDR-like rules and World Drug Index (WDI) rule. 2D structural models were drawn in ChemBioDraw Ultra version 12.0 (Cambridge Software) and SMILES of each compound were translated into molfile by online SMILES translator and structure file generator (National Cancer Institute) [16]. Molfile data was added into the database to predict drug-likeliness properties. Drug-likeliness score was calculated from Molsoft using SMILES as input.

#### ADMET profile prediction

ADMET profile of tributyltin (IV) compounds was predicted using PreADMET and SwissADME tools [17]. Molfiles created for each compound were added into the database and searched for ADMET properties. Degree of plasma protein binding (PPB) is categorized in preADMET as strongly bound if %PPB > 90% and weakly bound if %PPB < 90%. Blood brain barrier (BBB) penetration is presented as concentration ratio of steady-state of radiolabeled compounds in brain (C_brain_) and peripheral blood (C_blood_). Compounds are classified into high, middle and low absorbing into CNS with C_brain_/C_blood_ values of >2, 2-0.1 and <0.1 respectively. For assessing intestinal absorption, Caco-2 cell model categorizes compounds as low, middle and highly permeable corresponding to values <4 nm/sec, 4–70 nm/sec and >70 nm/sec respectively. SwissADME predicts BBB penetration and GI absorption by BOILED-Egg method [18]. It also classified compounds as targets of p-glycoprotein (p-gp) efflux, inhibitors of cytochrome P450 enzymes CYP2C9, CYP2C19, CYP2D6 and CYP3A4 and substrates for metabolism by CYP2D6 and CYP3A4. PreADMET anticipated toxicity of compound on models of Ames test with or without metabolic activation by S9 (rat liver homogenate) against strains of *Salmonella typhimurium* TA100 and TA1535 and rodent carcinogenicity constructed on National Toxicology Program and FDA US data on in-vivo 2 year carcinogenicity tests of mice and rats. Results are produced are positive or negative mutagenicity or carcinogenicity.

Metabolism of compounds via phase I and phase II reactions was predicted using MetaPrint2D React [19, 20] subjected to similarity to known sites of metabolism. It calculates normalized occurrence ration (NOR) indicating relative likelihood of metabolism, which occurs at a specific site in the molecule.

#### Molecular target prediction

Molecular targets were predicted by SwissTargetPrediction online tool [21]. Query molecules were drawn in 2D using the javascript-based molecular editor of ChemAxon and submitted to the database. SwissTargetPrediction envisages molecular targets based on chemical similarity (2D) and/or structural similarity (3D) among bioactive molecules. A threshold for 3D and 2D similarity values has been set to 0.75 and 0.45 respectively. Compounds having values beyond these thresholds are not listed.

### *In-vitro* screening

#### Cytotoxicity against THP-1 cell line

Cytotoxicity of tributyltin (IV) compounds against THP-1 cell line was determined by 3-(4,5-dimethylthiazol-2-yl)-2,5-diphenyl tetrazolium bromide (MTT) assay [22]. THP-1 cells were cultured in complete growth medium comprising RPMI-1640 (pH 7.4) supplemented with 2.2 g/L NaHCO_3_ and 10% v/v heat inactivated fetal bovine serum (HIFBS). An aliquot of 20 μL of samples in 1% DMSO in PBS and 180 μL of THP-1 cells were mixed in 96-well plate. Cells were added at an assay density of 1 × 10^4^ cells/mL whereas sample/standard was added to achieve the final concentration of 20 μg/mL. The plate was incubated at 37 °C for 72 h in humidified 5% carbon dioxide incubator (Panasonic, Japan MCO-18 AC-PE). Vincristine and 1% DMSO in PBS were used as positive and negative controls respectively. After incubation, 20 μL of pre-filter sterilized MTT solution (4 mg/mL in distilled water) was added in plates and incubated for 4 h at 37 °C in CO_2_ incubator. Later, colored formazan crystals were separated by removing supernatant and dissolved in 100 μL of DMSO. Reaction was allowed to stand for 1 h to ensure complete dissolution and absorbance was measured at 540 nm using microplate reader (Biotech USA, Elx 800).

#### Cytotoxicity assays against HepG2 cell line

Cytotoxicity was further evaluated against HepG2 cell line by sulforhodamine B (SRB) colorimetric assay [22]. Dulbecco's Modified Eagle Medium (DMEM) supplemented with 10% FBS, 100 μg/mL streptomycin sulfate, 100 IU/mL penicillin G sodium and 0.25 μg/mL amphotericin B was used to culture HepG2 cells in CO_2_ (5%) incubator (Panasonic, Japan MCO-18 AC-PE) at 37 °C. An aliquot of 20 μL (1% DMSO in PBS) of samples was added to 180 μL of cell culture at an assay density of 1x10^5^ cells/mL in 96-well plate. The plate was incubated at 37 °C for 72 h in CO_2_ incubator. Final concentration of compounds/standard was 20 μg/mL. Doxorubicin and 1% v/v DMSO in PBS ere used as positive and negative controls respectively. An equivalent number of cells in twelve wells of 96-well plate were incubated for 1 h at 37 °C and labeled as day zero control. Later, cells were fixed by adding 50 μL of cold 20% w/v TCA for 1 h at 4 °C. These were washed with tap water, air dried and stained with 50 μL of 0.057% w/v SRB in 1% v/v acetic acid for 30 min at room temperature. Cells were again washed with 1% v/v acetic acid, dried overnight and 200 μL of 10 mM Tris base (pH 10) was used to solubilize the bound dye for 1 h. Absorbance was measured at 515 nm using a microplate reader. Percentage of growth inhibition was calculated as: % inhibition = 100 – [(A_s_ – A_o_) / (A_n_ – A_o_) × 100], where, A_o,_ A_s_ and A_n_ are absorbance of day zero control, samples and negative control respectively. IC50 values were determined using 3-fold dilutions of the samples.

#### Cytotoxicity against isolated lymphocytes

Lymphocytes were isolated using previously described protocol with some modifications [23, 24]. Informed consent was obtained from volunteers and procedure was conducted according to international ethical guidelines after gaining approval from the ethical committee of the Quaid-i-Azam University. A volume of 3 mL of blood was obtained from a healthy donor by venipuncture and diluted (1:1) with PBS. It was layered over 2 mL Histopaque-1077 and centrifuged at 800 × g for 20 min. The buffy coat was aspirated into 5 mL of PBS and centrifuged at 350 rpm for 4 min to pellet the lymphocytes. The pellet was suspended in 1 mL of RPMI-1640 and cell density was adjusted to get 1 × 10^5^ cells/mL. For cytotoxicity determination, 20 μL of samples (20 μg/mL) or vincristine or 1% DMSO in PBS and 180 μL of lymphocyte suspension were incubated in 96-well plate at 37 °C for 24 h in humidified 5% carbon dioxide incubator (Panasonic, Japan MCO-18 AC-PE). Phytohaemagglutinin (PHA) was added in medium to stimulate lymphocyte growth. Afterwards, MTT assay was done as described above. IC50 values were determined using 3-fold dilutions of the samples.

#### Antileishmanial activity

Antileishmanial activity against the promastigotes of *Leishmania tropica* kwh 23 was evaluated by a quantitative colorimetric assay using 3-(4,5-dimethylthiazol-2-yl)-2, 5-diphenyl tetrazolium bromide (MTT) with minor modifications [25, 26]. *Leishmania tropica* parasites were cultured in Medium 199 supplemented with 10% FBS, 100 μg/mL streptomycin sulfate and 100 IU/mL penicillin G at 24 °C. A volume of 20 μL of extracts (100 μg/mL; DMSO ≤ 1% in PBS) and amphotericin B (0.33–0.004 μg/mL) were incubated with 180 μL of promastigotes at seeding density 2 × 10^6^ cells/mL in 96-well flat bottom plate at 25 °C for 72 h. Negative control wells contained 1% DMSO in PBS. All samples were run in triplicate. Afterwards, plates were incubated for 4 h at 24 °C with 20 μL of MTT (4 mg/mL in distilled water) to determine the cell viability. Supernatant from each well was carefully removed leaving behind formazan crystals. Colored formazan crystals were dissolved in 100 μL of DMSO by setting those aside for 1 h to ensure complete dissolution. Cell viability was estimated by measuring absorbance at 540 nm using microplate reader and percentage growth inhibition was calculated.

#### Determining photosensitizing effect against *leishmania*

Photosensitizing effect of tributyltin (IV) compounds was determined in three parallel groups [27]. Samples and controls were exposed for 15 min to sunlight (168 W/m^2^ of sun intensity) or sunlight with IR filter or dark conditions. The relative intensity of sunlight was measured by a CCD spectrometer (Ocean Optics, model HR4000). Later, plates were incubated at 25 °C for 72 h and cell viability was determined by MTT assay as given above. In this assay, samples were tested at concentrations showing less than 55% inhibition in primary antileishmanial assay to exclude false positive.

#### Determining ROS mediated antileishmanial activity

*Leishmania tropica* kwh 23 promastigotes were treated in three groups [27]. First group was exposed to samples along with 0.1 mM sodium azide (NaN_3_), second group to samples with 1 mM mannitol and third group comprised of all controls. In third group, promastigotes were treated in different wells with samples only (sample control), 1% DMSO in PBS only (negative control-I), amphotericin B only (positive control), 0.1 mM NaN_3_ only (negative control-II) and 1 mM mannitol only (negative control-III). Plates were incubated followed by MTT assay described above.

#### Protein kinase inhibition assay

Protein kinase inhibitory activity of the tributyltin (IV) compounds was measured against *Streptomyces* 85E strain using previously described protocol [28]. Culture of *Streptomyces* 85E was refreshed in tryptic soy broth and 100 μL of this was swabbed on sterile ISP4 medium under aseptic conditions. Sterile filter paper discs (6 mm) impregnated with 5 μL of samples (10 mg/mL in DMSO) or surfactin (2 mg/mL in DMSO; positive control) or DMSO (negative control) were placed on *Streptomyces* swabbed petri plate and incubated for 72-96 h at 30 °C. Diameter (mm) of zone of inhibition (ZOI) around all samples and controls was measured as clear zone (CZ) and bald zone (BZ). Clear zone represents *Streptomyces* growth inhibition whereas bald zone shows inhibition of hyphae and spore formation. BZ is indicative of PK inhibition.

#### Statistical analysis

Data is presented as Mean ± SD of respective parameters in triplicate analysis. One Way Analysis of Variance (ANOVA) was applied to measure significance of results. p < 0.05 was considered statistically significant. Analysis was done using GraphPad Prism 5.0 software.

## Results and discussion

### Drug-likeliness prediction

**Fig. 2:**
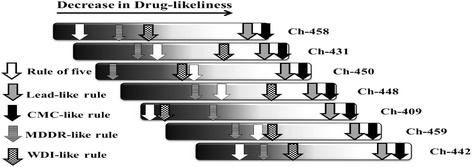
Drug-likeliness profile of tributyltin (IV) compounds: The color from black to white indicates decrease in compliance with drug-likeliness rules. Placement of arrows on compliance bar shows the extent to which a compound conform to respective rule depending upon number of the conditions fulfilled for each rule. It is predicted from PreADMET

Drug-likeliness prediction is important to optimize pharmaceutical and pharmacokinetic properties of compounds [29]. Tributyltin (IV) carboxylate compounds partially comply with selected rules of drug-likeliness. Ch-409 was in 90% cutoff range of WDI rule and complies with all conditions of Lipinski rule. Ch-458, 431, 448, 459 and 442 are mid-structures whereas Ch-450 is drug-like according to MDDR-like rule (Fig. 2). In practice, we have drugs in market that do not completely comply with drug-likeliness rules but are used due to their high beneficial effects for humans to treat cancer. For example, vincristine, which violates CMC-like, lead-like and Lipinski rules and complies with MDDR-like rule. Besides, cisplatin does not completely comply with any of the above rules. Drug-likeliness scores computed from Molsoft tool also indicate these compounds as moderately drug-like. Higher the score, greater the drug-likeliness conforming to available drugs [30]. Tributyltin (IV) compounds scores are -1.11, 0.42,–0.23, 0.15, 0.40,–0.49 and 0.27 for Ch-409, Ch-431, Ch-442, Ch-448, Ch-450, Ch-458 and Ch-459 respectively. On the other hand, cisplatin, vincristine, doxorubicin, amphotericin B, dactinomycin and sorafenib have drug-likeliness scores of -1.12, 1.38, 1.02, 0.9,–1.00 and 0.51 respectively. Thus, our compounds have comparable drug-likeliness profile with marketed drugs and are good drug candidates.

### ADMET prediction

ADME analysis done by PreADMET predicts that tributyltin (IV) carboxylate compounds have middle to high BBB permeability based on C_brain_/C_blood_ ratio of 0.942–11 whereas SwissADME predicts no BBB crossing since these compounds are also substrates for p-glycoprotein efflux (Table 1). P-gp is present on the apical surface of endothelial cells of blood brain barrier and impedes the entry of various drugs [31]. All compounds strongly bind with plasma protein except Ch-409 and have low to high GI absorption (caco-2 cells permeability 20.13–26.75 nm/sec). These compounds can be formulated into oral dosage form. Ch-409 is inhibitor of CYP2C19; Ch-450 and Ch-409 are inhibitors of CYP2C9; Ch-458, Ch-431, Ch-450, Ch-448, Ch-459 and Ch-442 are of CYP2D6 and Ch-450, Ch-409 and Ch-448 inhibit CYP3A4. No compound is substrate of CYP2D6 whereas all others except Ch-409, Ch-458 and Ch-442 are strong substrates for metabolism by CYP3A4. Our compounds were found mutagenic in Ames test either by point or frame-shift mutations (Table 1). Metabolic activation predicts mutagenicity of metabolites of Ch-458, Ch-450 and Ch-442. Carcinogenicity prediction was “out of range” in the database expect for Ch-409, which was not carcinogenic in rat model. Human ether a go-go-related (hERG) gene encodes cardiac potassium channels that mediate repolarization phase. Inhibition of these prolong QTc interval along with the risk of cardiac arrhythmias [32]. Our compounds show medium risk of hERG inhibition. Since, compounds show very high PPB and very low IC/LC50 values in in-vitro analysis; therefore, it is necessary to assess the concentration at which hERG inhibition risk is predominant.

**Table 1:** ADME prediction of tributyltin (IV) compounds

Samples		ADME Profile								Toxicity Profile					
	PPB (%)	BBB Permeability			Lipophilicity Consensus Log P_o/w_^a^	Water Sol^a^	GI Abs^a^	Caco-2 Cells (nm/sec)	P-gp Substrate^a^	Ames Test					hERG Inhibition
		PreADMET		Swiss Pred^a^						Pred	TA100		TA1535		
		(C_brain_/C_blood_)	Pred								+S9	-S9	+S9	-S9	
Ch-458	100	0.965	Middle	No	4.36	Poor	High	21.34	Yes	M	-ve	+ve	+ve	+ve	Medium
Ch-431	100	2.97	High	No	4.54	Poor	High	21.43	Yes	M	-ve	-ve	-ve	+ve	Medium
Ch-450	100	1.05	Middle	No	6.20	Insol	Low	26.75	Yes	M	-ve	-ve	+ve	+ve	Medium
Ch-448	100	0.971	Middle	No	7.58	Insol	Low	21.68	Yes	M	-ve	-ve	-ve	+ve	Medium
Ch-409	-ve	1.49	Middle	No	7.57	Insol	Low	22.28	Yes	M	-ve	+ve	-ve	-ve	Medium
Ch-459	100	0.942	Middle	No	4.93	Poor	High	22.03	Yes	M	-ve	-ve	-ve	+ve	Medium
Ch-442	100	1.14	Middle	No	4.35	Poor	High	21.34	No	M	-ve	-ve	+ve	+ve	Medium

^a^Data predicted from Swiss ADME tool. *Sol* = solubility; *abs* = absorption; *Insol* = insoluble; *M* = mutagen; -ve means no toxicity in Ames test; +ve means toxicity predicted in Ames test; *Pred* = prediction

**Fig. 3:**
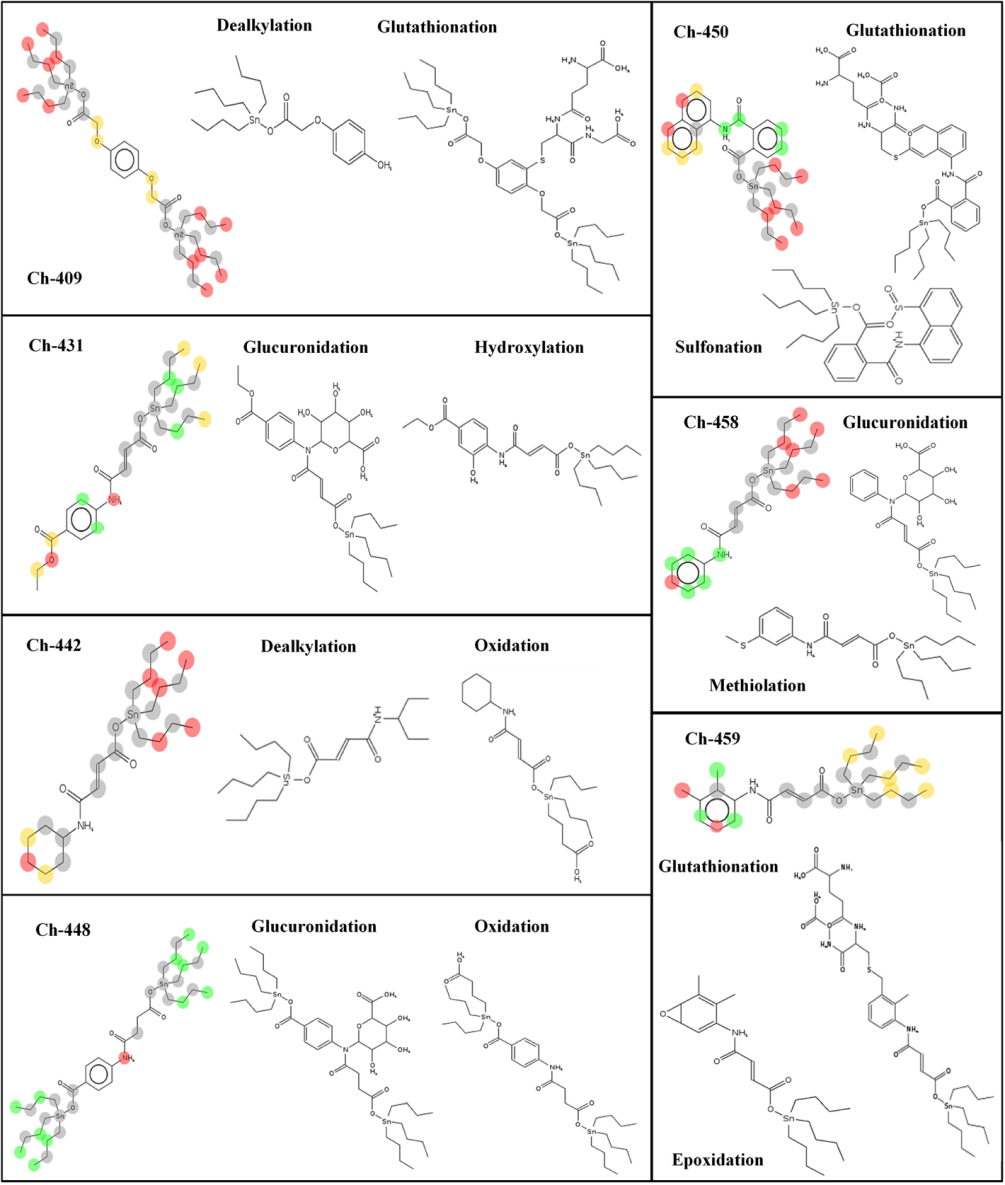
Predicted metabolic sites and metabolites of tributyltin (IV) compounds. Structures with colored circles indicate the metabolic sites predicted by MetaPrint2D React. Color codes are given based on normalized occurrence ratio. Red = 0.66 ≤ NOR ≤ 1.00; orange = 0.33 ≤ NOR < 0.66; green = 0.15 ≤ NOR < 0.33; white = 0.00 ≤ NOR < 0.15 and grey = little/no data. Two possible metabolites for each compound are given with NOR ≥ 0.33

Metabolic sites predicted by MetaPrint2D React are highlighted as red (0.66 ≤ NOR ≤ 1.00), orange (0.33 ≤ NOR < 0.66), green (0.15 ≤ NOR < 0.33), white (0.00 ≤ NOR < 0.15) and grey (little/no data) corresponding to NOR where high NOR means most frequently reported site in metabolism database (Fig. 3). It was found that besides dealkylation, hydroxylation and oxidation, all compounds are possibly metabolized by multiple phase I and phase II reactions. Ch-409 can undergo acetylcysteination and glutathionation; Ch-431 is metabolized by demethylation and glucuronidation; Ch-448 by glucuronidation; Ch-450 go through epoxidation, glutathionation, sulfonation, oxidative deamination and methoxylation whereas epoxidation, glucuronidation, methiolation, methoxylation are predicted for Ch-458 and epoxidation, glutathionation and methoxylation for Ch-459 (Fig. 3). Glucuronidation and glutathione metabolism of compounds make these hydrophilic and ionized at physiological pH. This also reduces their affinity with cellular target [33]. It is proposed that metabolism at multiple sites will excrete the compounds out of the body with less side effects of metabolites.

### Molecular target prediction

**Fig. 4:**
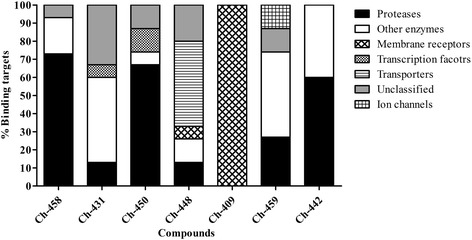
Predicted molecular targets for tributyltin (IV) compounds. Molecular target prediction is done by SwissTargetPrediction tool. Each bar represents the targets for each compound based on structural homology and binding potential of similar compounds in database. Unclassified shows the proteins that are not classified into any group

Molecular target prediction by SwissTargetPrediction tool provided possible interaction sites for tributyltin (IV) compounds. These include proteases, enzymes, transcription factor, receptors, ion channels and other proteins (Fig. 4). Although the probabilities of interaction with targets was low (range: 0.01–0.1) based on ChEMBL database; however, in-vitro analysis depicts good bioactivity profile of these compounds. This means that they interact strongly with cellular proteins and modify their functions to kill cancer cells and *leishmania* parasite. It was predicted that Ch-409 can bind with membrane receptors such as opioid (mu, kappa, delta) and nociceptin receptors. On the contrary, Ch-431 dominantly aims enzymes (47%) acetylcholinesterase, inosine-5'-monophosphate dehydrogenase 2 and tyrosyl-DNA phosphodiesterase 1; transcription factor (7%) hypoxia-inducible factor 1-alpha and other proteins (33%) including microtubule-associated protein tau and muscleblind-like protein 1, 2 and 3. Ch-442 was predicted to interact with serine proteases (60%) cathepsin G, granzyme B and H, chymotrypsin-C and chymotrypsin-like elastase family member 2A and enzymes (40%) peptidyl-prolyl cis-trans isomerase FKBP1A and B and peptidyl-prolyl cis-trans isomerase FKBP4 and 5. Ch-448 can regulate transporters (47%), for examples, excitatory amino acid transporters 1, 2, 3, 4 and 5. Other proteins include tyrosyl-DNA phosphodiesterase 1, FAD-linked sulfhydryl oxidase ALR; muscleblind-like proteins and membrane metallo-endopeptidase-like 1 (soluble form). Ch-450 targets 67% proteases (alpha-trypsin chain 1, trypsin 2 and 3, activation peptide fragment 1, urokinase-type plasminogen activator long chain A, tissue-type plasminogen activator, hepatocyte growth factor activator long chain, apolipoprotein-a), 7% other enzymes (enzyme FAD-linked sulfhydryl oxidase ALR), 13% transcription factors (oxysterols receptor LXR-alpha and beta) and 13% unclassified proteins (plectin, microtubule-associated protein tau). Ch-458 can dominantly control serine proteases (73%) including chymase, chymotrypsin-C, cathepsin G, Granzyme B and H, chymotrypsin-like elastase family member 1, 2A, 2B, 3A and 3B and neutrophil elastase as well as acetylcholinesterase, poly [ADP-ribose] polymerase 14 and microtubule-associated protein tau. Besides enzymes (74%; telomerase reverse transcriptase, cathepsin G, monoglyceride lipase), Ch-459 can also interact with ion channels (13%) like ATP-sensitive inward rectifier potassium channels 11, 1 and 8. It has been previously reported that some of these proteins are positive and negative regulators of cancer. For examples, microtubule-associated protein tau is associated with paclitaxel resistance in breast and gastric cancer patients [34, 35] and upregulated hepatocyte growth factor activator and trypsin like proteases in cancer tissues are involved with carcinogenesis and metastasis [36]. In contrast, muscleblind-like protein 1 is implicated in suppression of breast cancer metastatic colonization [37]; granzymes play key role in antitumor immunity [38] and FKBP1A is a target for anticancer drug rapamycin [39]. Predicted interaction of our compounds with previously reported proteins in cancer provides evidence for their beneficial role.

### Cytotoxicity of tributyltin (IV) compounds

Cytotoxicity of tributyltin (IV) compounds was quantified against HepG2, THP-1 cell lines and isolated lymphocytes at 20 μg/mL to compare effect at same concentration (Table 2). All compounds showed significantly higher (*p* < 0.05) cytotoxicity against HepG2 cell line except Ch-442. Ch-450 and Ch-459 exhibited significant cytotoxicity against THP-1 cells whereas activity of other compounds was lower than 50% at same concentration. On the contrary, Ch-409, Ch-459 and Ch-442 were toxic against normal isolated lymphocytes. However, IC50 values of these compounds for HepG2 cells were 10–100 times lower than those for isolated lymphocytes (Table 2). There was no significant cytotoxicity of other compounds against normal cells. This partially selective response against cancer cell lines is beneficial in targeting cancer cells whereas limiting damage to normal cells. Results are comparable with standard doxorubicin against HepG2 cells while lower in case of vincristine against THP-1 cells and lymphocytes. It can be seen that vincristine is significantly toxic against normal lymphocytes. Our compounds showed cytotoxicity against HepG2 cell line comparable to previously reported data on oxaliplatin and cisplatin [40]. On the contrary, lymphocyte cytotoxicity was more pronounced in carboplatin and cisplatin [23].

**Table 2:** Cytotoxicity and PK inhibition activity of tributyltin (IV) compounds

Samples	Cytotoxicity					Protein kinase inhibition assay				
	HepG2 (20 μg/mL)	IC50 (μg/mL)	THP-1 (20 μg/mL)	Isolated lymphocytes (20 μg/mL)	IC50 (μg/mL)	ZOI (mm) at 50 μg/disc		ZOI (mm) at 6.25 μg/disc		MIC (μg/disc)
	% growth inhibition		% growth inhibition			CZ	BZ	CZ	BZ	
Ch-458	98.7 ± 3.56*	1.58 ± 0.07	21.2 ± 1.57	14.8 ± 0.13ǂ	--	30 ± 1.2*	-	12 ± 1.25	31 ± 1.37*	0.195 ± 0.001
Ch-431	96.2 ± 2.23*	5.48 ± 0.16	42.7 ± 1.13	10.7 ± 1.17ǂ	--	31 ± 0.78*	33 ± 1.15*	10 ± 0.33	26 ± 0.16*	0.049 ± 0.002
Ch-450	98.5 ± 1.85*	2.81 ± 0.34	50.4 ± 2.63*	11.7 ± 0.05ǂ	--	32 ± 1.13*	-	-	28 ± 2.5*	0.195 ± 0.001
Ch-448	97.1 ± 2.34*	3.45 ± 0.63	43.1 ± 2.29	23.6 ± 1.07ǂ	--	31 ± 1.05*	-	-	25 ± 1.75*	0.781 ± 0.002
Ch-409	99.5 ± 3.15*	0.08 ± 0.001	19.4 ± 1.41	55.6 ± 2.32*ǂ	15.18 ± 0.75ǂ	30 ± 1.75*	31 ± 1.5*	12 ± 0.19	30 ± 1.17*	0.781 ± 0.002
Ch-459	96.5 ± 4.66*	0.62 ± 0.005	52.5 ± 0.99*	60.7 ± 1.68*	5.78 ± 0.34ǂ	35 ± 0.99*	-	-	30 ± 0.24*	0.391 ± 0.004
Ch-442	12.8 ± 1.37	--	44.6 ± 1.18	55.4 ± 1.74*ǂ	14.9 ± 0.25	40 ± 1.17*	-	10 ± 0.74	29 ± 0.78*	0.391 ± 0.006
Vincristine	--	--	100 ± 0.0*	76.3 ± 3.65*	6.66 ± 0.09	--	--	--	--	--
Doxorubicin	97.21 ± 1.14*	5.36 ± 0.75	--	--	--	--	--	--	--	--
Surfactin (10 μg/mL)	--	--	--	--	--	-	23 ± 1.98*	--	--	--
1% DMSO with PBS	0	--	0	0	--	--	--	--	--	--
DMSO	--	--	--	--	--	0	0	0	0	--

Data is Mean ± SD; *n* = 3; (--) indicates not applied; *ZOI* = zone of inhibition of Streptomyces; (-) shows no *ZOI*; CZ = clear zone of inhibition; *BZ* = bald zone of inhibition, *MIC* = minimum inhibitory concentration; (*) means *p* < 0.05 compared with negative control; (ǂ) means data is significantly different from HepG2 results

It was reported that carboxylate derivatives of organotin such as phenylacetate, benzoate and cinnamates were found effective against tumor cell lines. Some of the di-n-butyltin compounds were proved more potent than cisplatin [41]. In line with this, our results are comparable with previous study where different organotin (IV) carboxylate compounds revealed significant anticancer activity against kidney fibroblast (BHK-21) and lung carcinoma (H-157) cell lines [10]. This indicates prospective of tributyltin (IV) carboxylate compounds as potential candidates for anticancer drug development. Further screening of compounds used in present study is recommended against a range of cancer and normal cell lines to evaluate their efficacy and selectivity. Several reports presented that there is no well-defined mechanism by which organotin compounds can interact with cancer cells. Their intercalation with phosphodiester backbone of DNA is reported, altering intracellular breakdown of phospholipids of endoplasmic reticulum [42, 43]. Organotin compounds may also bind with membrane proteins, cellular kinases, ATPase or glycoproteins [44]. We have found these compounds as protein kinase inhibitors, which can be one of the mechanisms for cytotoxicity. However, detailed mechanism of action of these compounds is to be elucidated.

### Antileishmanial activity

Tributyltin (IV) compounds demonstrated highly significant (*p* < 0.05) antileishmanial activity with >90% lethality and very low LC50s ranging from 0.954 ± 0.158 μg/mL to 0.078 ± 0.002 μg/mL (Table 3). Lowest LC50 was observed by Ch-431 that appears equipotent to the standard amphotericin B. It has been reported that sodium stibogluconate showed IC50s of 64 μg/mL and 22 μg/mL against axenic log-phase promastigotes and cellular metacyclic promastigotes respectively [45]. Our compounds appear to be more potent than sodium stibogluconate. Compounds were further tested for their photocatalytic activity by exposing to sunlight, sunlight with IR filter and dark conditions. There was 0–32% toxicity against* leishmania* when exposed to light and dark conditions without samples. Compounds showed no significant photosensitizing effect after normalizing the data indicating that their activity is independent of light conditions. Samples were further tested in the presence of ROS scavengers NaN_3_ and mannitol, which scavenge singlet oxygen [46] and hydroxyl radicals [47] respectively. ROS are important molecules in controlling *leishmania* infection that are generated by macrophages during phagocytosis of parasite [48]. It was observed that antileishmanial activity of compounds was reduced by 16.38%–34.38% in the presence of NaN_3_ whereas it was reduced by 15–38.2% in the presence of mannitol as compared with 16% and 10% decline for amphotericin B in the presence of NaN_3_ and mannitol respectively. Ch-442 showed selective toxicity against *leishmania* with 95.8 ± 1.16% lethality as compared with 12.8 ± 0.37% and 44.6 ± 0.18% cytotoxicity against HepG2 and THP-1 cells respectively. Moreover, antileishmanial L50 (0.088 ± 0.009 μg/mL) is approximately 15 times lower than IC50 (14.9 ± 0.25 μg/mL) against isolated lymphocytes. Activity of Ch-442 was reduced by 22% and 38.2% in the presence of NaN_3_ and mannitol respectively. These results indicate that ROS production by compounds is one of the mechanisms for antileishmanial effect.

**Table 3:** Antileishmanial activity of tributyltin (IV) compounds

Samples	Antileishmanial activity		ROS scavenging at 20 μg/mL		Photosensitizing effect			
	% growth inhibition at 20 μg/mL	LC50 μg/mL	% growth inhibition					
			NaN3 (0.1 mM)	Mannitol (1 mM)	Control	Sunlight	Sunlight with IR filter	Dark
Ch-458	97.8 ± 1.23*	0.256 ± 0.064	73.5 ± 2.78ǂ	60.3 ± 1.5ǂ	35.2 ± 1.78	42.7 ± 1.35	30.25 ± 0.17	36.7 ± 0.74
Ch-431	97.5 ± 2.75*	0.078 ± 0.002	66.2 ± 2.99ǂ	59.6 ± 0.19ǂ	50.7 ± 1.25	53.2 ± 1.55	47.5 ± 0.99	51.5 ± 1.35
Ch-450	97.4 ± 1.35*	0.361 ± 0.017	75.4 ± 1.75ǂ	62.8 ± 2.74ǂ	24.5 ± 1.63	32.12 ± 1.28	20.7 ± 0.43	26.34 ± 0.15
Ch-448	96.5 ± 2.67*	0.576 ± 0.088	77.9 ± 3.25ǂ	68.9 ± 0.75ǂ	54.65 ± 2.75	49.7 ± 0.89	52.9 ± 1.17	50.16 ± 1.19
Ch-409	97.5 ± 1.98*	0.954 ± 0.158	81.12 ± 1.63	82.5 ± 1.53	52.05 ± 1.98	57.63 ± 1.99	47.5 ± 1.56	56.9 ± 0.02
Ch-459	97.9 ± 3.99*	0.323 ± 0.002	63.52 ± 0.86ǂ	76.7 ± 1.17ǂ	20.1 ± 0.59	25.5 ± 1.75	30.14 ± 1.47	22.7 ± 1.05
Ch-442	95.8 ± 2.16*	0.088 ± 0.009	73.8 ± 1.36ǂ	57.6 ± 0.78ǂ	46.1 ± 0.56	40.5 ± 0.12	49.25 ± 0.63	44.18 ± 1.16
Amphotericin B	100 ± 0.0*	0.044 ± 0.001	84 ± 2.05	90 ± 1.85	--	--	--	--
1% DMSO with PBS	0	0	0	0	0	0	0	0

Data is Mean ± SD. (--) means not applied; (*) means *p* < 0.05 as compared with negative control. (ǂ) *p* < 0.05 compared with untreated control. Concentrations for photosensitizing assay are 0.55 μg/mL for Ch-409 and Ch-448; 0.092 μg/mL for Ch-431, Ch-442, Ch-458, Ch-459 and Ch-450

### Structure-activity relationship

Tributylstannyl carboxylate is the primary moiety responsible for cytotoxicity of these compounds. We compared all compounds with Ch-458, which has double bond at position 2, phenylamino ring and one tributylstannyl group with an IC50 of 1.58 μg/mL against HepG2 cells. Replacing the oxo-phenylaminobutanoic acid ligand with phenylenebis(oxy) diacetic acid has enhanced the potency of Ch-409 by 19-fold providing maximum cytotoxicity with lowest IC50 value. Dimethyl substitution at phenylamino ring in Ch-459 augmented its potency by 2.54-fold compared to Ch-458. On the contrary, substitutions of naphthalene-1-ylcarbamoyl, ethoxycarbonyl at position 4 on phenylamino group and missing double bond at position 2 reduced potency of compounds Ch-450, Ch-431 and Ch-448 by 1.77, 3.46 and 2.18-folds respectively. However, presence of cyclohexyl ring instead of phenyl may have hindered binding affinity of Ch-442 to target proteins in HepG2 cells causing no cytotoxicity.

In case of THP-1 cells, addition of naphthalene (Ch-450) and dimethylphenyl (Ch-459) might have enhanced binding of compounds to specific targets in THP-1 cells that vary from HepG2 and isolated lymphocytes. Nevertheless, the cytotoxicity is only around 50% at concentration of 20 μg/mL. Since, proteins and cellular components may vary between normal and cancer cells; therefore, activity of our compounds was pronounced against HepG2 cells with little/no effect against isolated lymphocytes. Substitutions of cyclohexyl ring (Ch-442), dimethylphenyl (Ch-459) and phenylenebis(oxy) diacetic acid (Ch-409) showed cytotoxicity against isolated lymphocytes. It is possible that these compounds gained access to biomarkers similar in normal and cancer cells. Since, Ch-459 showed cytotoxicity in all three cell types used; therefore, presence of dimethylphenyl group appears to be an important modulator of cytotoxicity.

Compounds were also highly cytotoxic against *leishmania* promastigotes. Ch-409 with phenylenebis(oxy) diacetic acid ligand was least potent among all compounds with LC50 0.954 μg/mL. Substitution of ethoxycarbonyl at position 4 on phenylamino group (Ch-431) gained maximum access to specific leishmania proteins and was 12.23-fold potent than Ch-409. Cyclohexyl moiety in Ch-442 showed targeted affinity for leishmania specific proteins or other biomolecules allowing selective cytotoxicity behavior with 10.84-fold higher potency than Ch-409 and 1.12-fold lower than Ch-431. Similarly, respective substitutions in Ch-458, Ch-459, Ch-450 and Ch-448 reduced potency by 3.28, 4.14, 4.62 and 7.38-folds compared with Ch-431. Reduction of a double bond at position 2 drastically decreased potency of Ch-448. Thus, tributylstannyl carboxylate backbone is highly cytotoxic against *leishmania* promastigotes and ligand modifications on it caused potency variations. Our results indicate dissimilarity in compounds’ behavior against cancer cells, normal cells and *leishmania*. It depicts that these have different molecular targets as well as variable affinity against same biomolecules. This is supported by our *in-silico* molecular target prediction where both variable and overlapping interaction site have been predicted. Thus, these compounds have multimode cytotoxicity with potential for varied diseases.

### Protein kinase inhibition activity

Protein kinases are essential modulators of cellular regulations and physiological processes [49]. Irregular activation of these kinases constitutes oncogenic signals. Cytoplasmic (Abl) or transmembrane (PDGFR, EGFR) protein kinases found in many cancer cells are targets for anticancer drugs to induce cytotoxicity [50]. Thus, we assessed inhibition of protein kinases as possible mechanism of cytotoxicity in our study.

Being highly cytotoxic, initially clear zones were observed; however, compounds provided bald zones at lower doses with no cytotoxicity. Bald zone specify protein kinase inhibition by obstructing aerial hyphae formation whereas clear zones indicate complete inhibition of *Streptomyces* growth. Lowest MIC for PK inhibition was shown by Ch-431 (Table 2). MIC for PK inhibition is much lower than IC50 for HepG2 cytotoxicity and is somewhat comparable with LC50 for antileishmanial activity. It means that these compounds are effective in targeting protein kinase enzymes and other associated factors at lower doses that those which cause cytotoxicity.

Molecular target prediction predicted enzymes as likely targets. Although, *in-silico* target prediction shows little data on binding probability with kinases; however, we experimentally proved this fact that tributyltin (IV) carboxylate compounds used in our study significantly inhibited (*p* < 0.001) protein kinase activity in *Streptomyces*. Streptomyces requires protein kinase activity of RamC to convert pre-Sap B to Sap B, a surfactant important for aerial hyphae formation [51]. It has been found that RamC is a membrane associated receptor kinase [51], which supports binding of all our compounds with this motif including Ch-409 whose major targets are predicted to be receptors only. This indicates significance of tributyltin (IV) compounds in protein kinase inhibition that may be a reason for cytotoxicity in cancer cells and *leishmania*. Oncogenesis is mediated either directly or indirectly by transmembrane or cytoplasmic tyrosine/serine-threonine kinases [52]. Furthermore, MAPK MAPK-like kinases, cyclin-dependent kinase (Cdk) and glycogen synthase kinase 3 are implicated in growth, differentiation and infectivity of *leishmania* parasite [53]. Thus, our data suggests molecular level studies to further explore exact mechanism of these compounds.

## Conclusions

Tributyltin (IV) carboxylate compounds are contenders of anticancer and antileishmanial drug development. *In-silico* analysis shows these compounds partially drug-like, permeable across GI membrane and blood brain barrier and mutagenic with possible risk of hERG inhibition. High PPB and rate of metabolism will provide very low free drug concentration in plasma that may reduce the risk of side effects, which can be further managed using targeted drug delivery system. Our new compounds are highly cytotoxic against HepG2 cells and *leishmania* parasite with lower cytotoxicity against normal isolated lymphocytes. These significantly inhibit protein kinase with very low IC50 values. These compounds can be evaluated further based on their risk benefit ratio.

## Abbreviations

ADMET: Absorption, distribution, metabolism, elimination and toxicity
BZ: Bald zone
CZ: Clear zone
NOR: Normalized occurrence ration
ZOI: Zone of inhibition.
